# IL-33 Acts to Express Schaffer Collateral/CA1 LTP and Regulate Learning and Memory by Targeting MyD88

**DOI:** 10.1155/2017/2531453

**Published:** 2017-09-24

**Authors:** Tomoyuki Nishizaki

**Affiliations:** Innovative Bioinformation Research Organization, Kobe 651-1223, Japan

## Abstract

Interleukin-33 (IL-33) is recognized to transmit a signal through a heterodimeric receptor complex ST2/interleukin-1 receptor accessory protein (IL-1RAcP) bearing activation of myeloid differentiation factor 88 (MyD88). High-frequency stimulation to the Schaffer collateral induced long-term potentiation (LTP) in the CA1 region of hippocampal slices from wild-type control mice. Schaffer collateral/CA1 LTP in IL-33-deficient mice was significantly suppressed, which was neutralized by application with IL-33. Similar suppression of the LTP was found with MyD88-deficient mice but not with ST2-deficient mice. In the water maze test, the acquisition latency in IL-33-deficient and MyD88-deficient mice was significantly prolonged as compared with that in wild-type control mice. Moreover, the retention latency in MyD88-deficient mice was markedly prolonged. In contrast, the acquisition and retention latencies in ST2-deficient mice were not affected. Taken together, these results show that IL-33 acts to express Schaffer collateral/CA1 LTP relevant to spatial learning and memory in a MyD88-dependent manner and that the LTP might be expressed through an IL-1R1/IL-1RAcP-MyD88 pathway in the absence of ST2.

## 1. Introduction

**Figure 1:**
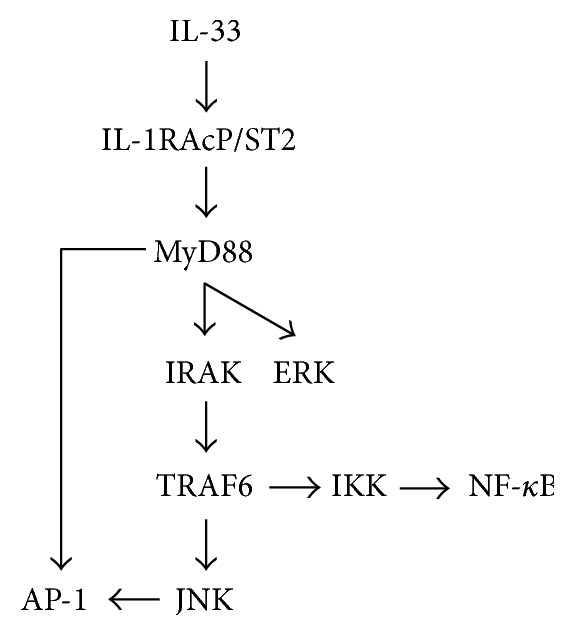
A schematic diagram of an IL-33 signaling pathway.

Interleukin-33 (IL-33), a member of the IL-1 family [1], is expressed in a variety of cell types such as immune cells, including mast cells, macrophages, and dendritic cells, and nonimmune cells, including endothelial, epithelial, and smooth muscle cells and fibroblasts [2]. IL-33 serves as not only a proinflammatory cytokine but also a nuclear factor [3, 4]. Extracellularly secreted IL-33 binds to ST2 and activates a heterodimeric receptor complex, comprising ST2 and interleukin-1 receptor accessory protein (IL-1RAcP) on the plasma membrane, to recruit and activate myeloid differentiation factor 88 (MyD88), followed by activation of interleukin-1 receptor-associated kinase (IRAK) and tumor necrosis factor (TNF) receptor-associated factor 6 (TRAF6) [5] (Figure 1). Intracellular IL-33, alternatively, is localized in the nucleus due to its association with heterochromatin via a helix-turn-helix motif within the N-terminal part, where IL-33 acts as a transcriptional repressor [6, 7] (Figure 1).

Several lines of evidence have pointed to the role of IL-33 in the pathogenesis of Alzheimer's disease (AD). Expression of TNF-*α* and IL-1*β*, that are potent inducers of IL-33 [8], is upregulated in the AD brain [9]. Moreover, strong expression of IL-33 and ST2 is found in the vicinity of amyloid plaques and neurofibrillary tangles [10]. A contradictory finding is that IL-33 transcript levels are decreased in the AD brain [11]. Intriguingly, IL-33 administration reverses synaptic plasticity impairment and memory deficits in APP/PS1 double-transgenic mice, an animal AD model [12].

The present study was conducted to assess the effects of IL-33 on synaptic plasticity and spatial learning and memory and to understand its relevant signaling pathway. The results show that IL-33 acts to express Schaffer collateral/CA1 long-term potentiation (LTP) relevant to spatial learning and memory in a MyD88-dependent manner. The results also suggest that excessive IL-1RAcP under the ST2-lacking conditions might bind to IL-1R1, allowing activation of MyD88, and therefore that an IL-1R1/IL-1RAcP-MyD88 pathway might be involved in the expression of the LTP in the absence of ST2.

## 2. Materials and Methods

All procedures have been approved by the Animal Care and Use Committee at Hyogo College of Medicine (Nishizaki's previous laboratory) and were carried out in compliance with the National Institutes of Health Guide for the Care and Use of Laboratory Animals.

### 2.1. Mice

*IL-33* ^−/−^ Balb/c mice, *ST2*^−/−^ C57/Bl6 mice, and *MyD88*^−/−^ Balb/c mice were kindly provided by Professor T. Yoshimoto (Hyogo College of Medicine, Nishinomiya, Japan) [13, 14]. Respective littermate wild-type mice were used as controls.

### 2.2. Field Excitatory Postsynaptic Potential (fEPSP) Recording

fEPSP recording was carried out by the method as previously described [15–18]. Briefly, fEPSPs were monitored from the CA1 area of hippocampal slices by electrically stimulating the Schaffer collateral at 0.033 Hz in artificial cerebrospinal fluid (ACSF) (in mM: 117 NaCl, 3.6 KCl, 1.2 NaH_2_PO_4_, 1.2 MgCl_2_, 2.5 CaCl_2_, 25 NaHCO_3_, and 11.5 glucose) oxygenated with 95% O_2_ and 5% CO_2_ at 34°C. ACSF was also used as a recording electrode-filling solution.

The parameters for high-frequency stimulation (HFS) to induce LTP were four trains with an intertrain interval of 200 ms, and each train consisted of ten 30 s 200 Hz pulses.

### 2.3. Water Maze Test

Water maze test was carried out by the method as previously described [17–19]. Briefly, mice were placed in the pool filled with muddy water containing India ink, and time from start to escape onto the platform (acquisition latency) was measured. Two trials were carried out for a day, and the second trial began 30 s after the end of the first trial. Mice received the task for 8 consecutive days, and the mean latency from 2 consecutive days was calculated. Seven days later, two trials were performed again, and time from start to escape onto the platform (retention latency) was monitored as retention latency.

Swim speed was calculated from time and move distance from start to escape onto the platform in each trial.

### 2.4. Statistical Analysis

Statistical analysis was carried out using unpaired *t*-test and analysis of variance (ANOVA) followed by Fisher's protected least significant difference (PLSD) test.

## 3. Results

### 3.1. IL-33 Is Required for Expression of Schaffer Collateral/CA1 LTP

**Figure 2:**
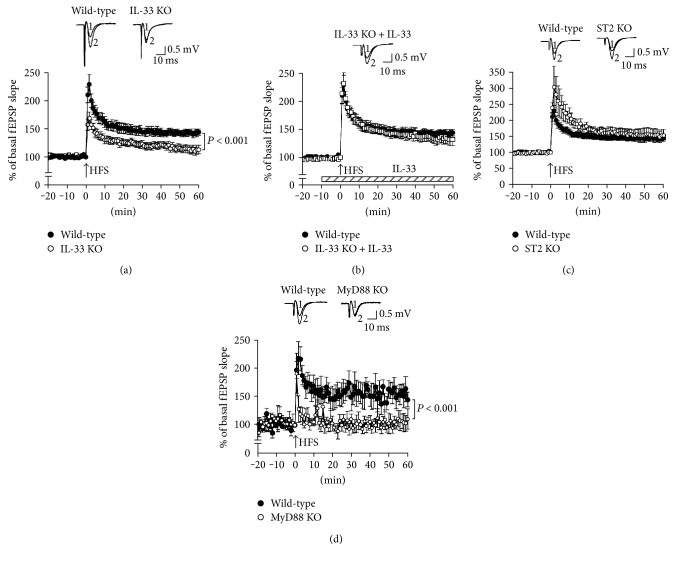
Schaffer collateral/CA1 LTP. fEPSPs were monitored in the CA1 region of hippocampal slices from wild-type mice (wild-type), IL-33-deficient mice (IL-33 KO) in the absence and presence of IL-33 (1 ng/mL) (a, b), ST2-deficient mice (ST2 KO) (c), and MyD88-deficient mice (MyD88 KO) (d) before and after HFS. In the graphs, each point represents the mean (±SEM) percentage of basal EPSP slope (0 min) (*n* = 6–13 independent experiments). *P* values, ANOVA followed by Fisher's PLSD test.

Initially, fEPSPs were monitored in the CA1 region of hippocampal slices from wild-type control mice by electrically stimulating the Schaffer collateral commissural pathway. HFS enhanced fEPSP slopes to 150–230% of basal levels in wild-type control mice, being evident at 60 min after HFS; in other words, HFS induced Schaffer collateral/CA1 LTP in wild-type control mice (Figure 2(a)). HFS-induced potentiation of fEPSP slopes in IL-33-deficient mice was significantly lesser as compared with the potentiation in wild-type control mice (Figure 2(a)). This implies that Schaffer collateral/CA1LTP is suppressed by IL-33 deficiency. Amazingly, suppression of the LTP in IL-33-deficient mice was neutralized by adding IL-33 (1 ng/mL) (Figure 2(b)). This indicates that IL-33 is required for expression of the LTP.

IL-33 binds to ST2 and activates an ST2/IL-1RAcP-MyD88 pathway (Figure 1). Unexpectedly, no significant effect on Schaffer collateral/CA1 LTP was obtained with ST2-deficient mice, although the potentiation in the initial phase was decreased (Figure 2(c)). This indicates that the LTP could be expressed even in the absence of ST2.

Schaffer collateral/CA1 LTP in MyD88-deficient mice, on the other hand, was abolished (Figure 2(d)). This indicates that MyD88 is a key factor for expression of the LTP.

### 3.2. IL-33 Regulates Spatial Learning and Memory

**Figure 3:**
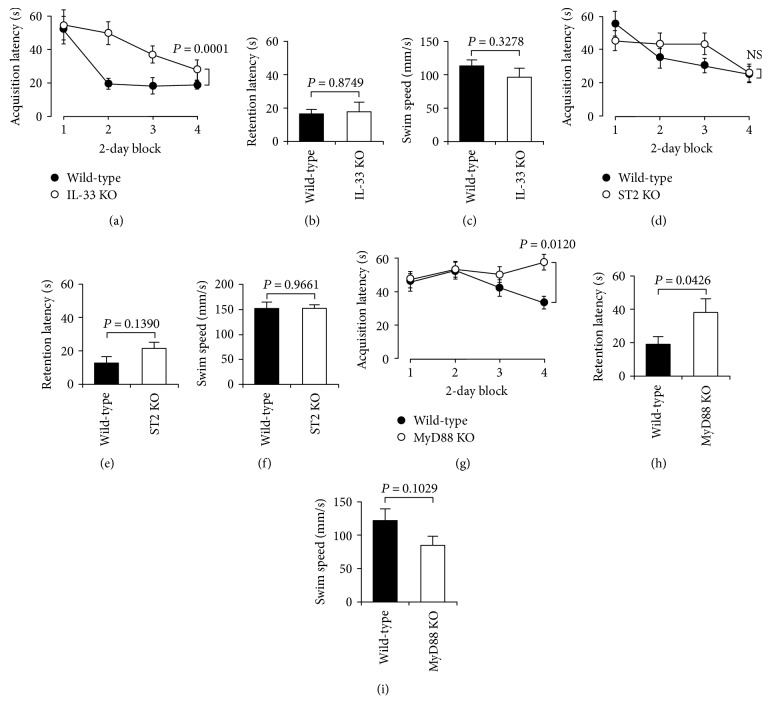
Water maze test. The water maze task was performed two trials per day for 8 consecutive days, and the acquisition latency was measured in wild-type mice (wild-type), IL-33-deficient mice (IL-33 KO), ST2-deficient mice (ST2 KO), and MyD88-deficient mice (MyD88 KO). Seven days later, the retention latency was measured. In addition, swim speed was calculated in each trial. (a, d, g) In the graphs, each point represents the mean (±SEM) acquisition latency from 2 consecutive days (*n* = 6-7 independent mice). *P* values, ANOVA followed by Fisher's PLSD test. NS: not significant. (b, e, h) In the graphs, each column represents the mean (±SEM) retention latency (*n* = 6-7 independent mice). *P* values, unpaired *t*-test. (c, f, i) In the graphs, each column represents the mean (±SEM) swim speed (*n* = 6-7 independent mice). *P* values, unpaired *t*-test.

In the water maze test, the acquisition latency in IL-33-deficient mice was significantly prolonged as compared with that in wild-type control mice (Figure 3(a)), but there was no significant difference in the retention latency between IL-33-deficient mice and wild-type control mice (Figure 3(b)). This indicates that IL-33 is required for regulation of spatial learning. The swim speed in IL-33-deficient mice was at the level similar to that in wild-type control mice (Figure 3(c)). This interprets that the prolonged acquisition latency in IL-33-deficient mice is not due to the slowdown of the swim speed.

The acquisition and retention latencies in ST2-deficient mice were not different from the latencies in wild-type control mice (Figures 3(d) and 3(e)). In addition, there was no significant difference in the swim speed between ST2-deficient mice and wild-type control mice (Figure 3(f)). These results suggest that ST2 is not implicated in the regulation of spatial learning and memory.

Both the acquisition and retention latencies in MyD88-deficient mice were significantly longer than those in wild-type control mice (Figures 3(g) and 3(h)), without affecting swim speed (Figure 3(i)). This indicates that MyD88 plays a critical role in the regulation of spatial learning and memory. Overall, these results raise the possibility that IL-33 regulates spatial learning and memory by targeting MyD88.

## 4. Discussion

Several avenues of evidence have pointed to the role of the cytokines IL-6 and IL-18 in synaptic transmission and synaptic plasticity such as LTP and long-term depression (LTD), a cellular model of learning and memory. The IL-6 mRNA is upregulated during induction of *N*-methyl-D-aspartate (NMDA) receptor-dependent LTP [20, 21]. IL-6 inhibits hippocampal LTP through a tyrosine kinase/extracellular signal-regulated kinase (ERK) pathway [22–24]. IL-18 stimulated glutamate release from presynaptic terminals and enhances responses of postsynaptic *α*-amino-3-hydroxy-5-methyl-4-isoxazole propionic acid (AMPA) receptors, leading to facilitation of hippocampal synaptic transmission [15]. Moreover, IL-18 regulates motor activity, anxiety, and spatial learning without affecting synaptic plasticity [18].

In the present study, Schaffer collateral/CA1 LTP in IL-33-deficient mice was suppressed, and the suppression was neutralized by adding IL-33. This implies that IL-33 is required for expression of the LTP. In the water maze test, the acquisition latency in IL-33-deficient mice was significantly prolonged, indicating impairment of spatial learning. Taken together, IL-33 appears to enhance learning ability by expressing the LTP.

It is shown that IL-33 rises in AD brain cells and that amyloid *β*_1–42_ upregulates IL-33 in cultured mouse astrocytes [10]. Strangely, the IL-33 mRNA levels in the hippocampus of 5xFAD mice, an animal model of AD, were significantly lower than the levels for wild-type control mice, but otherwise, the IL-33 protein levels in the hippocampus of 5xFAD mice were significantly higher than the levels for wild-type control mice [25]. This indicates that in spite of the decline of the IL-33 mRNA transcription, translation of IL-33 protein is accelerated in the AD brain. Cognitive impairment in AD, in the light of the findings, might be attributed to decrease of IL-33 protein in association with downregulation of the IL-33 mRNA in the earlier stage of AD, but to restore it, translation of IL-33 protein might be stimulated as a compensatory function in the chronic stage.

IL-33 binds to ST2 and activates an ST2/IL-1RAcP-MyD88 pathway [5]. Activated MyD88 activates: (i) c-Jun-N-terminal kinase (JNK), a mitogen-activated protein kinase (MAPK), along an IRAK/TRAF6 axis; (ii) nuclear factor-*κ*B (NF-*κ*B) along an IRAK/TRAF6/I*κ*B kinase (IKK) axis; (iii) ERK, a MAPK; and (iv) activator protein 1 (AP-1) (Figure 1).

For ST2-deficient mice, Schaffer collateral/CA1 LTP was not suppressed and the acquisition and retention latencies in the water maze test were not affected. This suggests that ST2 is not implicated in the expression of the LTP and the regulation of spatial learning and memory.

**Figure 4:**
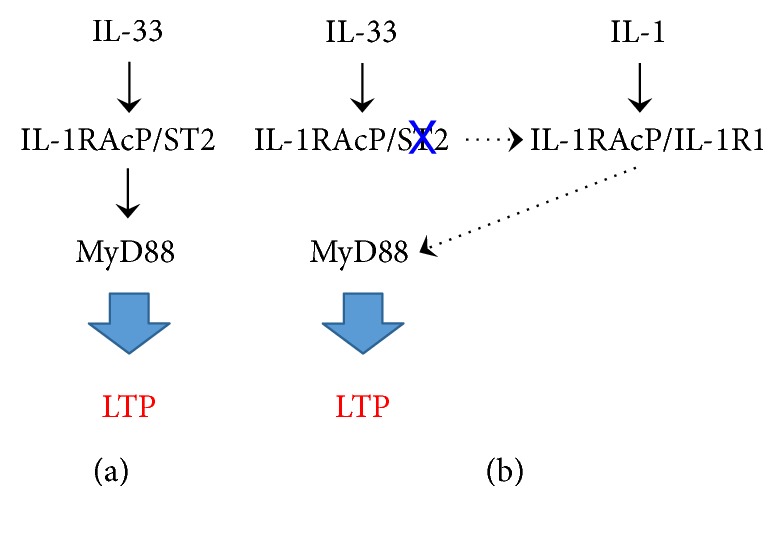
Plausible pathways for IL-33-induced LTP in the presence (a) and absence (b) of ST2.

For MyD88-deficient mice, Schaffer collateral/CA1 LTP was abolished and both the acquisition and retention latencies in the water maze test were significantly prolonged. This indicates that MyD88 is a key factor to express the LTP and regulate spatial learning and memory. Overall, the results of the present study lead to a conclusion that IL-33 acts to express Schaffer collateral/CA1 LTP relevant to spatial learning and memory by targeting MyD88 (Figure 4(a)).

The big question addressed is why Schaffer collateral/CA1 LTP and spatial learning and memory were not affected in ST2-deficient mice. A plausible explanation for this is that excessive IL-1RAcP under the ST2-lacking conditions might bind to IL-1R1, allowing activation of MyD88, thereby expressing Schaffer collateral/CA1 LTP and enhancing spatial learning and memory. Consequently, the LTP might be expressed though an IL-1R1/IL-1RAcP-MyD88 pathway in the absence of ST2 (Figure 2(b)).

## 5. Conclusion

The results of the present study show that IL-33 expresses Schaffer collateral/CA1 LTP and regulates spatial learning and memory through an IL-33-(ST2/IL-1RAcP)-MyD88 pathway in the presence of ST2 or through an IL-1-(IL-1R1/IL-1RAcP)-MyD88 pathway in the absence of ST2. This may extend our understanding about the role of IL-33 in cognitive functions.
